# Effect of Low-Dose MDCT and Iterative Reconstruction on Trabecular Bone Microstructure Assessment

**DOI:** 10.1371/journal.pone.0159903

**Published:** 2016-07-22

**Authors:** Felix K. Kopp, Konstantin Holzapfel, Thomas Baum, Radin A. Nasirudin, Kai Mei, Eduardo G. Garcia, Rainer Burgkart, Ernst J. Rummeny, Jan S. Kirschke, Peter B. Noël

**Affiliations:** 1 Department of Diagnostic and Interventional Radiology, Klinikum rechts der Isar, Technische Universität München, Munich, Germany; 2 Department of Orthopedic Surgery, Klinikum rechts der Isar, Technische Universität München, Munich, Germany; 3 Section of Neuroradiology, Klinikum rechts der Isar, Technische Universität München, Munich, Germany; 4 Chair for Biomedical Physics, Physik-Department, Technische Universität München, Garching, Germany; Chongqing University, CHINA

## Abstract

We investigated the effects of low-dose multi detector computed tomography (MDCT) in combination with statistical iterative reconstruction algorithms on trabecular bone microstructure parameters. Twelve donated vertebrae were scanned with the routine radiation exposure used in our department (standard-dose) and a low-dose protocol. Reconstructions were performed with filtered backprojection (FBP) and maximum-likelihood based statistical iterative reconstruction (SIR). Trabecular bone microstructure parameters were assessed and statistically compared for each reconstruction. Moreover, fracture loads of the vertebrae were biomechanically determined and correlated to the assessed microstructure parameters. Trabecular bone microstructure parameters based on low-dose MDCT and SIR significantly correlated with vertebral bone strength. There was no significant difference between microstructure parameters calculated on low-dose SIR and standard-dose FBP images. However, the results revealed a strong dependency on the regularization strength applied during SIR. It was observed that stronger regularization might corrupt the microstructure analysis, because the trabecular structure is a very small detail that might get lost during the regularization process. As a consequence, the introduction of SIR for trabecular bone microstructure analysis requires a specific optimization of the regularization parameters. Moreover, in comparison to other approaches, superior noise-resolution trade-offs can be found with the proposed methods.

## Introduction

Osteoporosis is defined as a skeletal disorder characterized by compromised bone strength predisposing an individual to an increased risk for fracture [1]. Osteoporotic fractures not only considerably reduce quality of life but are also associated with an increased mortality. Due to the aging population, the prevalence of osteoporosis and accordingly the incidence of osteoporotic fractures are expected to increase. Therefore, osteoporosis is classified as a public health problem. The socio-economic burden is expected to rise dramatically, e.g. in the United States from $17 billion in 2005 by almost 50% until 2025 [2].

The assessment of osteoporosis associated fracture risk has traditionally relied on dual-energy X-ray absorptiometry (DXA) for the measurement of bone mineral density (BMD) at the spine and hip. However, BMD values of patients with and without osteoporotic fractures statistically overlap [3–5]. Therefore, the Fracture Risk Assessment Tool (FRAX) has been introduced which uses easily obtainable clinical risk factors to estimate a 10-year fracture probability in order to provide a better clinical guidance for treatment decisions. Furthermore, considerable research effort has been undertaken to develop non-invasive imaging techniques focusing on the assessment of cortical and trabecular bone microstructure to improve fracture risk predictions [6]. These imaging techniques include high-resolution magnetic resonance imaging (MRI), high-resolution peripheral quantitative computed tomography (hr-pQCT) and high-resolution multi-detector computed tomography (MDCT) [6–8]. However, hr-pQCT and MRI are limited to peripheral sites and cannot be applied to the spine, which is one of clinically most relevant fracture sites. It has been demonstrated that MDCT-based bone microstructure parameters and finite element models (FEM) improved the prediction of bone strength beyond BMD [6, 9–12]. However, in-vivo MDCT imaging for bone microstructure analysis and FEM at the spine is associated with an effective dose of estimated 3 mSv for one vertebra [13]. This dose is close to the diagnostic reference value of medically indicated radiation exposure and not acceptable for longitudinal assessment of fracture risk and therapy monitoring. Unfortunately these measurements are—so far—not clinical routine and thus are limited to research trails. Clearly a reduction of radiation exposure is needed; however, such a reduction would cause a significant increase in image noise and reduction in diagnostic image quality. On the contrary, advanced reconstruction algorithms, such as iterative approaches, are well known to reduce image noise and improve the diagnostic quality [14–19].

The purpose of our study was to investigate the effects of low-dose MDCT and in-house developed fully statistical iterative reconstruction (SIR) algorithms on trabecular bone microstructure parameters. We hypothesized that trabecular bone microstructure parameters assessed by low-dose MDCT in combination with SIR algorithms adequately predict vertebral bone strength in-vitro as compared to bone microstructure measurements based on established standard-dose MDCT protocols.

## Materials and Methods

### Specimens

The human donors had dedicated their bodies for educational and research purposes to the local Institute of Anatomy prior to death, in compliance with local institutional and legislative requirements. Written informed consent was obtained from the donors. The study was reviewed and approved by the local institutional review boards (Ethikkommission der Fakultät für Medizin der Technischen Universität München, Munich, Germany). Twelve vertebrae between thoracic vertebra 5 and 12 were harvested from three fresh human cadavers (one woman aged 74 years and two men aged 46 and 62 years). The donors had no history of pathological bone changes other than osteoporosis, i.e. bone metastases, hematological, or metabolic bone disorders. The surrounding muscle, fat tissue, and intervertebral discs were completely removed. Each vertebra was embedded in resin (Rencast Isocyanat and Polyol, Huntsman Group, Bad Säckingen, Germany) up to 2 mm above respectively below their vertebral endplates for the purpose of biomechanical testing. The resin fixation was performed with parallel alignment of the upper and lower endplate of the vertebrae with the outer surface of the resin chock to guarantee strict axial loading conditions of the vertebrae during the uniaxial biomechanical test. Specimens were stored in a refrigerator at +10°C between preparation and testing in sealed plastic bags. Mechanical testing was performed at room temperature (+19°C, moisture ∼55%). All vertebrae were in sodium chloride solution at least 3 h before imaging to prevent air artifacts. During imaging the vertebrae were sealed in vacuum plastic boxes filled with sodium chloride solution.

### MDCT Imaging

MDCT imaging was performed with a 64-row MDCT scanner (Somatom Definition AS, Siemens Medical Solutions, Erlangen, Germany). Each vertebra stored in a plastic box filled with sodium chloride solution as outlined above was placed in a plastic container filled with water to simulate an in-vivo examination. For calibration purposes, a reference phantom with a bone-like and a water-like phase (Osteo Phantom, Siemens Medical Solutions Erlangen, Germany) was placed in the scanner bed beneath the plastic container. The routine radiation exposure used in our department (standard-dose, SD) and a low-dose protocol (LD) were applied to each vertebra. The SD and LD protocol had both a pitch factor of 0.8, tube voltage of 120 kV, and tube current of 220 mA and 70 mA, respectively. Voxel size and slice thickness were 300 x 300 *μ*m^2^ and 600 *μ*m in both protocols. That amounts to an estimated effective dose of 2.5 mSv for SD and 0.79 mSv for LD for one vertebra.

### Image Reconstruction

All datasets were reconstructed with filtered backprojection (FBP) and SIR. The implementation of SIR is based on separable paraboloidal surrogates (SPS) with ordered subsets [20]. A Poisson distribution is used to model the noise of the measurement. Paraboloidal surrogates are used to find the maximum of the log-likelihood:

$$\begin{array}{c} L\left(\mu \right)=\sum_{i}y_{i}\text{log}\left(b_{i}e^{-{\left[A\mu \right]}_{i}}\right)-b_{i}e^{-{\left[A\mu \right]}_{i}}, \end{array}$$ (1)

where the sum runs over all measured rays *i*. *y* is the measurement, *A* is the system matrix, *μ* is the image and *b* is the intensity that would be recorded if the object was absent. Ideally, this maximization is performed iteratively until the result converges.

Statistical iterative reconstruction algorithms are ill-posed in nature and thus a penalty function (regularization) is necessary to control image noise. We employed

$$\begin{array}{c} \Delta (\mu )=L(\mu )-\beta R(\mu ), \end{array}$$ (2)

where *R* is a roughness penalty and the parameter *β* controls the strength of the penalty. The roughness penalty can be expressed by:

$$\begin{array}{c} R\left(\mu \right)=\sum_{j}\sum_{k\in N_{j}}v_{k}\psi \left(\mu_{j}-\mu_{k}\right), \end{array}$$ (3)

where *N*_*j*_ is the set of neighbors of pixel *j*. *v*_*k*_ is a weight depending on the order of the neighboring pixel *k* and *ψ* is the potential function. We used Lange’s potential function [21]:

$$\begin{array}{c} \psi (x)=\delta [|t/\delta |-log(1+|t/\delta |)]. \end{array}$$ (4)

This potential function belongs to the group of edge-preserving regularization. *δ* is a threshold defining what intensity differences are smoothed. The number of iterations was selected by comparing the intermediate results after each iteration. In our case, ideal image quality was reached after 15 iterations. This number was used for all SIR in this work.

SIR was performed without regularization (SIR w/o reg.) and with different regularization parameters. Regularization parameters were chosen by extensive testing of the data from one osteoporotic and one healthy bone. We started with values *β* = 0.0001 and *δ* = 0.00001 and increased them stepwise by a factor of ten until we reached *β* = 0.1, *δ* = 0.01 and having used all different (*β*, *δ*)-combinations in between. Trabecular microstructure parameters were assessed for each reconstruction and compared to the parameters from SD-FBP by computing the average relative error. Additionally, images were compared visually. Based on the extensive testing, best results in this work were reconstructed with *β* = 0.001, *δ* = 0.0001. Moreover, we present results for reconstructions with *β* = 0.1, to show the effect of a stronger regularization, and *β* = 0 (SIR w/o reg.) to show the effect of no regularization.

### MDCT Image Analysis

One person performed all steps of the MDCT image analysis. MDCT images were loaded into an in-house developed program based on IDL (Interactive Data Language, Research Systems, Bolder, CO, USA). According to QCT-based BMD measurements [22], the most central third of all slices displaying the vertebra equidistant to its endplates were identified. Then, circular regions of interest (ROIs) were manually placed in the ventral half of the vertebra in the selected slices of the MDCT images. The circular ROI had a diameter of 10 mm. Furthermore, ROIs were drawn in the phases of the calibration phantom in the MDCT images. BMD in the ROIs was calculated by converting the pixel attenuations in Hounsfield Units [HU] into calcium hydroxyapatite [mg/cm^3^] by using the calibration phantom. Afterwards, MDCT images were binarized to calculate trabecular bone microstructure parameters. An optimized global threshold was applied to all MDCT images. Similar to previous studies, 200 mg/cm^3^ calcium hydroxyapatite was identified as optimized global threshold [9, 23]. Four morphometric parameters were calculated in the ROIs in analogy to standard histomorphometry using the mean intercept length method [24]: bone volume divided by total volume (BV/TV), trabecular number (TbN; [mm^−1^]), trabecular separation (TbSp; [mm]), and trabecular thickness (TbTh; [mm]). Parameters were labeled as apparent (app.) values, since they cannot depict the true trabecular structure due to the limited spatial resolution [13]. In addition, fractal dimension (FD) as texture measurement of the trabecular bone structure was determined in the MDCT images using a box counting algorithm [25].

### Biomechanical Testing

**Fig 1:**
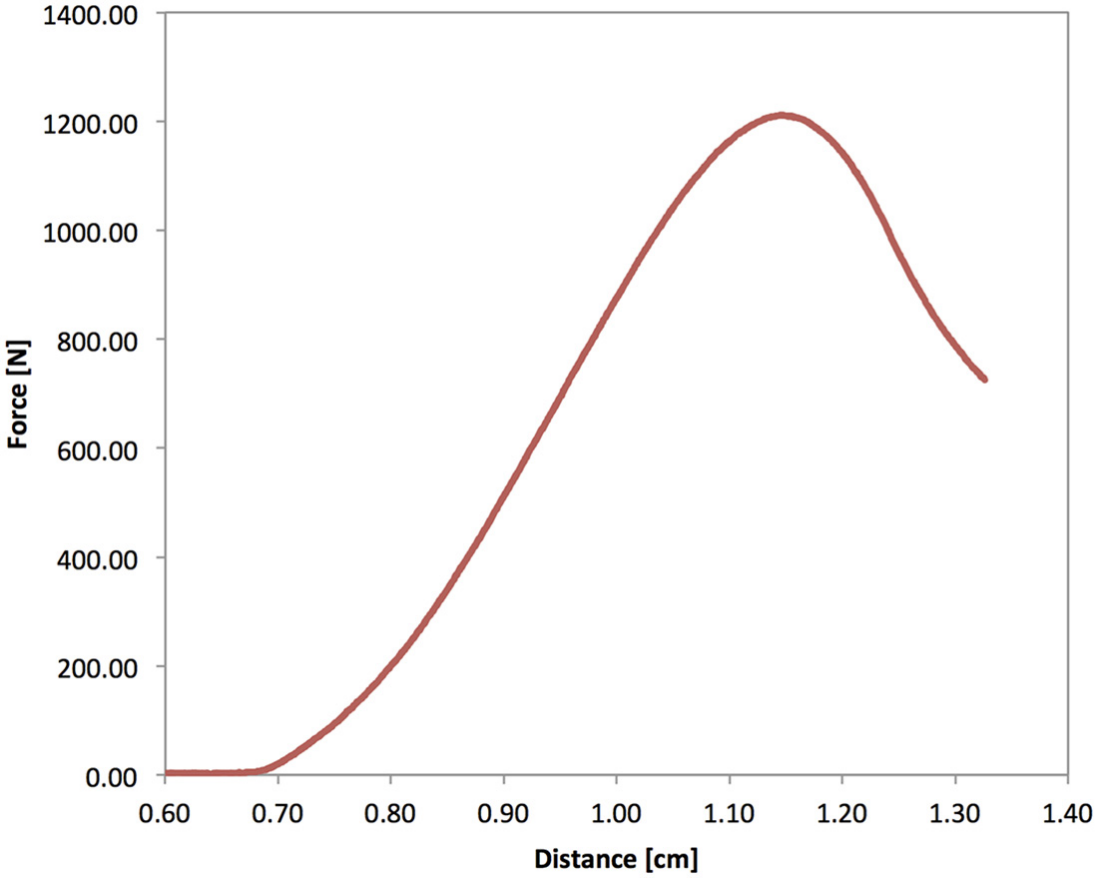
Sample load-displacement curve. Load-displacement curve from the biomechanical testing of a vertebra (ID: OPS004_1). FL was defined as the first peak of the load-displacement curve with a subsequent drop in force >10%.

**Fig 2:**
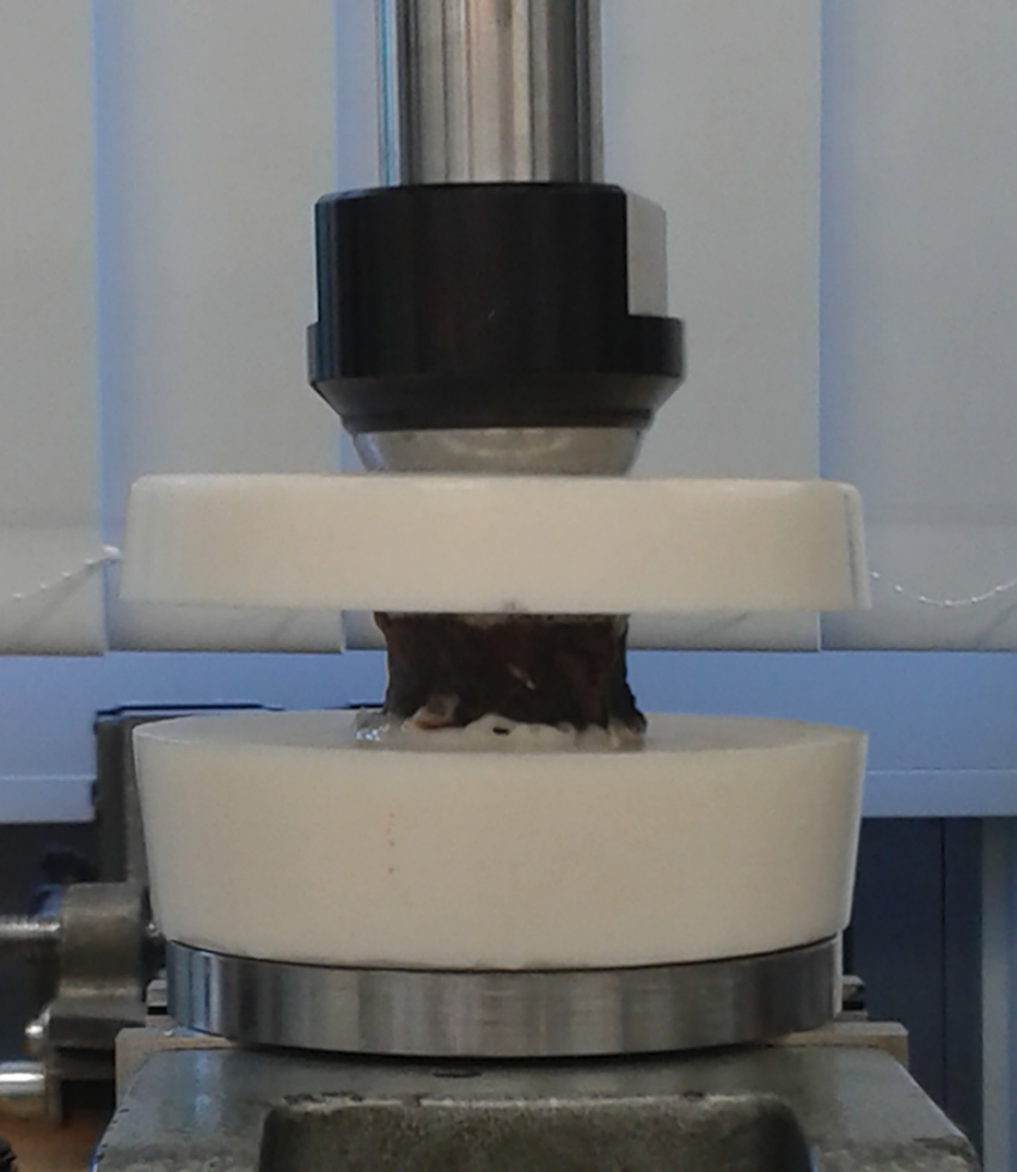
Setup of the biomechanical testing. Setup of the biomechanical testing to determine FL. The vertebra is fixed in a mechanical testing system.

The biomechanial testing was performed similar to previous studies [23, 26, 27]. The resin embedded vertebrae were fixed in a mechanical testing system (Wolpert Werkstoffprüfmaschinen AG, Schaffhausen, Switzerland). A ball joint was used for the mechanical testing to guarantee axial loading. Ten pre-conditioning cycles with uniaxial tension-compression up to a load between 10 N and 400 N with a rate of 5 mm/min were applied. Then, a monotonic, uniaxial compression was performed at the same rate. The load-displacement curve was recorded and vertebral fracture load (FL) was defined as the first peak of the load-displacement curve with a subsequent drop in force >10% (Fig 1). The setup for biomechanical testing is shown in Fig 2.

### Statistical Analysis

The statistical analysis was performed with SPSS software package (SPSS, Chicago, IL, USA). All tests were done using a two-sided 0.05 level of significance. Mean and standard deviation of the trabecular bone microstructure parameters were calculated. The Kolmogorov-Smirnov test showed for most parameters a significant difference from normal distribution (*p* < 0.05). Therefore, correlations between trabecular bone microstructure parameters and FL were evaluated with the Spearman’s rank correlation coefficient (*r*). The Friedman test was used to compare the whole set of microstructure parameters. Moreover, the parameters assessed from each reconstruction were compared to the parameters as assessed with SD-FBP using the Wilcoxon rank sum test.

## Results

The absolute values of the trabecular microstructure parameters are noted together with the measured FL and BMD in the supporting information (S2 Table).

**Fig 3:**
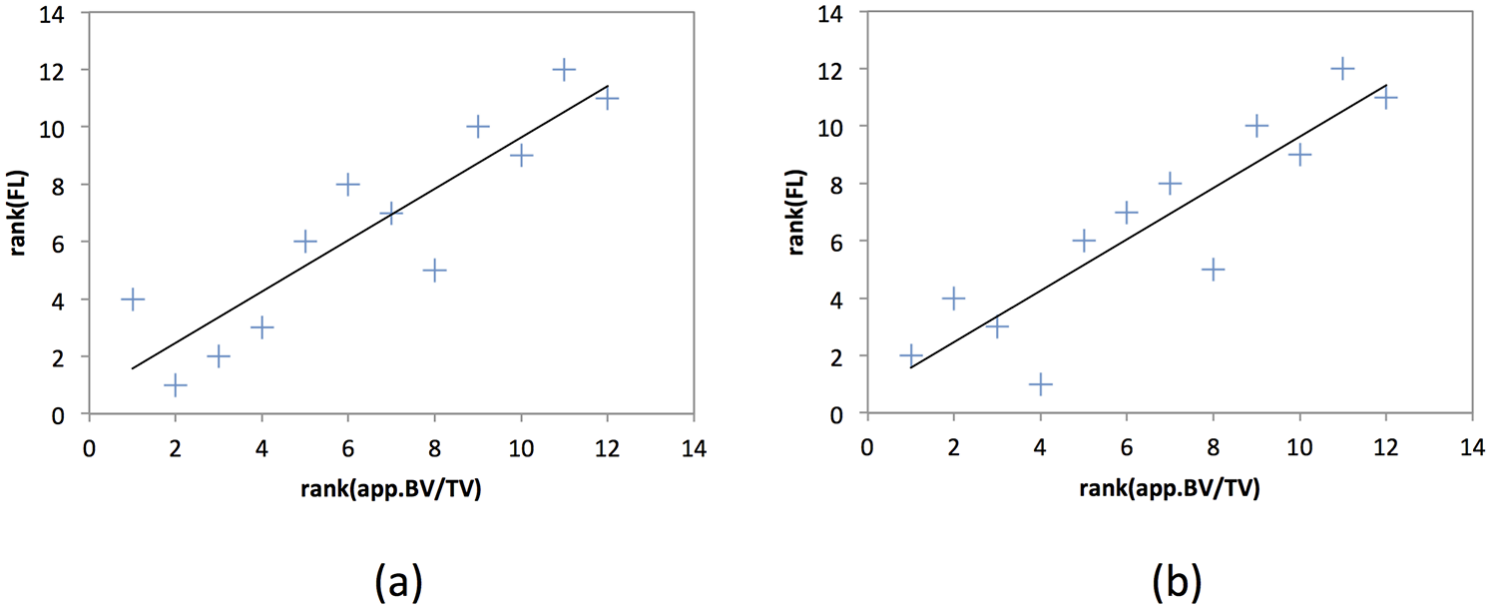
Correlation app.BV/TV versus FL. Sample correlation plots for app.BV/TV vs. FL ((a) SD-FBP, (b) LD-SIR *β* = 0.001, *δ* = 0.0001). Note that the correlation plots show the ranked values for app.BV/TV and FL because we used Spearman’s rank correlation coefficient.

According to the Friedman test, the absolute values of the trabecular bone microstructure parameters as assessed with SD-FBP, LD-FBP and LD-SIR with and without regularization were significantly different (*p* < 0.05). Trabecular bone microstructure parameters showed significant correlations with FL in the range of *r* = 0.88 − 0.91 (SD-FBP), *r* = 0.62 − 0.85 (LD-FBP), *r* = 0.69 − 0.93 (LD-SIR w/o reg.), *r* = 0.58 − 0.81 (LD-SIR *β* = 0.1, *δ* = 0.0001) and *r* = 0.84 − 0.91 (LD-SIR *β* = 0.001, *δ* = 0.0001) (*p* < 0.05; Table 1). Fig 3 visualises the correlation between app.BV/TV and FL exemplarily for our reference reconstruction (SD-FBP; Fig 3a) and for the best LD result (LD-SIR *β* = 0.001, *δ* = 0.0001; Fig 3b).

**Table 1:** Correlation coefficients versus FL.

			LD-SIR		
	SD-FBP	LD-FBP	w/o reg.	*β* = 0.1, *δ* = 0.0001	*β* = 0.001, *δ* = 0.0001
app.BV/TV	0.90 (*p* < 0.001)	0.85 (*p* < 0.001)	0.93 (*p* < 0.001)	0.81 (*p* = 0.002)	0.90 (*p* < 0.001)
app.TbN	0.88 (*p* < 0.001)	0.77 (*p* = 0.003)	0.87 (*p* < 0.001)	0.67 (*p* = 0.020)	0.91 (*p* < 0.001)
app.TbSp	-0.90 (*p* < 0.001)	-0.85 (*p* < 0.001)	-0.90 (*p* < 0.001)	-0.67 (*p* = 0.020)	-0.91 (*p* < 0.001)
app.TbTh	0.91 (*p* < 0.001)	0.85 (*p* = 0.001)	0.92 (*p* < 0.001)	0.58 (*p* = 0.046)	0.84 (*p* = 0.001)
FD	0.89 (*p* < 0.001)	0.62 (*p* = 0.031)	0.69 (*p* = 0.014)	0.65 (*p* = 0.024)	0.89 (*p* < 0.001)

Spearman’s rank correlation coefficient *r* between FL and trabecular bone microstructure parameters as assessed with SD and LD protocols and reconstructed with FBP and SIR. All parameters correlated significantly (*p*-value <0.05) with FL.

Comparing all parameters to the values assessed with SD-FBP using the Wilcoxon rank sum test showed significant differences for the following values (*p* < 0.05; Table 2): TbN, FD for LD-FBP; TbN, TbSp, TbTh, FD for LD-SIR with *β* = 0.1, *δ* = 0.0001. There was no significant difference between SD-FBP and LD-SIR w/o reg. and between SD-FBP and LD-SIR with *β* = 0.001, *δ* = 0.0001.

**Table 2:** Wilcoxon rank sum test against standard-dose FBP data.

		LD-SIR		
	LD-FBP	w/o reg.	*β* = 0.1, *δ* = 0.0001	*β* = 0.001, *δ* = 0.0001
app.BV/TV	*p* = 0.603	*p* = 0.564	*p* = 0.057	*p* = 0.403
app.TbN	*p* < 0.001	*p* = 0.863	*p* = 0.001	*p* = 0.436
app.TbSp	*p* = 0.194	*p* = 0.544	*p* = 0.001	*p* = 0.341
app.TbTh	*p* = 0.729	*p* = 0.544	*p* = 0.017	*p* = 0.470
FD	*p* = 0.046	*p* = 0.285	*p* = 0.014	*p* = 0.236

Wilcoxon rank sum test of trabecular bone microstructure parameters as assessed with different reconstructions versus SD-FBP. *p*-values <0.05 indicate significant differences.

**Fig 4:**
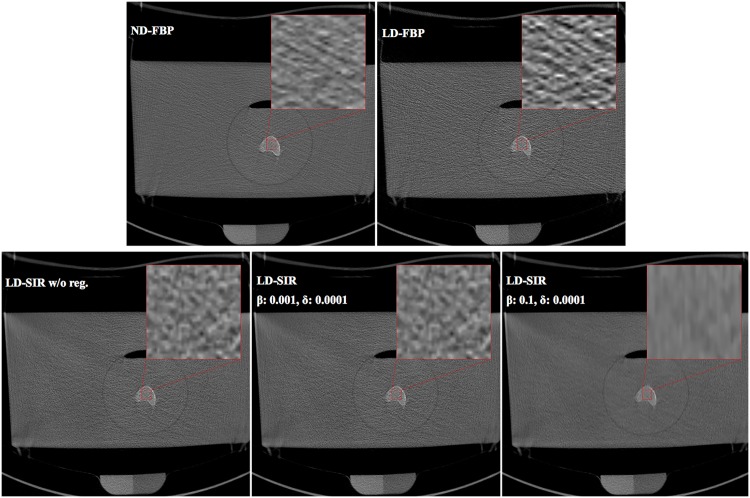
Comparison of CT images from different doses. MDCT images of a representative vertebra in a water bath to simulate an in-vivo examination. For visual comparison of the bone microstructure, it is magnified by a factor of 10. Scans were performed with SD and LD protocols and images were reconstructed with FBP and SIR. (Level 300 HU, window 2000 HU).

Fig 4 shows images acquired with LD and SD reconstructed with different settings and algorithms. Clearly, images reconstructed with FBP are noisier than images reconstructed with SIR. The results of Table 2 show that we can reach the same quality as with SD-FBP for the structure parameters using LD-SIR. But the regularization parameters have to be chosen carefully. A relative strong regularization (*β* = 0.1, *δ* = 0.0001) resulted in significant differences for the microstructure parameters.

## Discussion

The purpose of this study was to investigate the diagnostic quality, at two different radiation dose levels, of FBP and SIR for the calculation of trabecular bone microstructure parameters. Radiation exposure associated with CT diagnostics is a relevant challenge in the day-to-day clinical routine. The number of CT examinations is increasing with the rising number of indications and applications. The growing challenge of fulfilling the ALARA principle remains the major task for clinicians as well as for the research community. For example, at the same time microstructure parameters and FEM improve the prediction of bone strength. Thus, with advanced reconstruction algorithms becoming clinically available clinicians have the opportunity to introduce new metrics (such as microstructure assessment) while keeping the radiation dose at a low level.

Trabecular bone microstructure parameters significantly correlated with FL for all reconstructions. However, the absolute values showed significant differences. One can observe that parameters assessed for LD-FBP caused the majority of absolute values to drift towards the range of healthy bone. Hence, we can come to the conclusion that image noise is partly counted as bone-tissue. This behavior of LD (noisy) FBP reconstructions could lead to wrong results in the structure analysis. These observations lead to the conclusion that by using SIR, the noise level decreases while maintaining the anatomical and pathological information of bones.

Previous studies performed in-vitro vertebral MDCT imaging and reconstruction based on SD-FBP. Baum et al. [23] reported correlation coefficients up to *r* = 0.79 between bone microstructure parameters and FL, and Dall’Ara et al. [27] up to *r* = 0.89 between finite element models and FL. We observed correlation coefficients in a similar range.

The results of the Wilcoxon rank sum tests reveal that LD-SIR yield in equal prediction of the bone strength as SD-FBP. However, the results revealed a strong dependency on the regularization strength. We demonstrated that regularization might corrupt the microstructure analysis. The ultra small trabecular bone microstructure is just small detail in the images and will therefore be lost when applying too much regularization. As a consequence, the introduction of SIR for trabecular bone microstructure analysis requires a specific optimization of the regularization parameters and number of iterations.

Automated parameter selection for iterative reconstructions is still subject of ongoing research. In this work, we chose the parameters for iterative reconstruction based on extensive testing on two datasets—one with high and one with low bone strength.

On this note, for SIR type algorithms such a parameter search has only to be done once for a specific diagnostic question and radiation dose level. An optimization for different radiation exposures could be done with a low-dose simulation tool [28, 29]. For the future, with such a tool not only the parameter optimization but additionally the lowest possible dose level can be determined.

One drawback in our study design is the relatively small sample size of twelve vertebrae from three different donors. However, the goal of this work was to investigate the effect of LD protocols and SIR on the measurement of trabecular bone microstructure parameters. For this technical driven purpose of this study the sample size may be considered as sufficient.

With respect to radiation exposure reduction our work illustrated the possibility to employ advanced reconstruction algorithms to assess structural bone information under low dose situations. Other investigators have illustrated that there is a spectrum of other techniques which reduces dose within a given range [30]. For example with regard to osteoporosis assessment of the spine, one could foresee to use region-of-interest techniques to reduce the dose in the peripheral [31–33]. Further, the introduction of single photon-counting detectors will enable the clinician to reduce dose and improve the spatial resolution [34, 35], which could generate high interest for osteoporosis screeneing. Further, the additional information, which are made available via spectral imaging, can be used to improve task driven reconstruction algorithms [36].

## Conclusion

It was shown that trabecular bone microstructure parameters as assessed by LD-SIR significantly correlated with vertebral bone strength. The parameters were not significantly different to parameters assessed by SD-FBP. Therefore, LD protocols and SIR algorithms may allow the clinical use of MDCT-based trabecular bone microstructure analysis at the spine with an acceptable radiation exposure. This would allow for the future to improve fracture risk prediction and therapy monitoring in the context of osteoporosis. However, absolute values of the trabecular bone microstructure parameters are dependent on the MDCT protocol and reconstruction algorithm. This has to be considered when translating advanced algorithms into the clinical area. In conclusion, our results showed that SIR with properly tuned parameters—for regularization and number of iterations—allows the usage of MDCT based trabecular bone microstructure assessment at significantly reduced radiation dose.

## Supporting Information

S1 Table Abbreviations and acronyms. (PDF)

S2 Table Measured trabecular bone microstructure parameters and fracture load for each vertebra. Vertebrae with the same 3-digit number are from the same donor. The FD parameter value for LD-SIR *β* = 0.1, *δ* = 0.0001 of vertebra OPS004_2 could not be determined. The correlation FD vs. FL for LD-SIR *β* = 0.1, *δ* = 0.0001 was computed without consideration of vertebra OPS004_2.
(PDF)

## References

[1] NIH. NIH Consensus Development Panel on Osteoporosis Prevention, Diagnosis, and Therapy, March 7–29, 2000: highlights of the conference. South Med J. 2001;94:569–573. 11440324

[2] Burge R, Dawson-Hughes B, Solomon DH, Wong JB, King A, Tosteson A. Incidence and economic burden of osteoporosis-related fractures in the United States, 2005–2025. Journal of bone and mineral research. 2007;22(3):465–475. 10.1359/jbmr.061113 17144789

[3] Kanis JA, Melton LJ, Christiansen C, Johnston CC, Khaltaev N. The diagnosis of osteoporosis. Journal of bone and mineral research. 1994;9(8):1137–1141. 10.1002/jbmr.5650090802 7976495

[4] Schuit S, Van der Klift M, Weel A, De Laet C, Burger H, Seeman E, et al Fracture incidence and association with bone mineral density in elderly men and women: the Rotterdam Study. Bone. 2004;34(1):195–202. 10.1016/j.bone.2003.10.001 14751578

[5] Siris ES, Chen YT, Abbott TA, Barrett-Connor E, Miller PD, Wehren LE, et al Bone mineral density thresholds for pharmacological intervention to prevent fractures. Archives of Internal Medicine. 2004;164(10):1108–1112. 10.1001/archinte.164.10.1108 15159268

[6] Link TM. Osteoporosis imaging: state of the art and advanced imaging. Radiology. 2012;263(1):3–17. 10.1148/radiol.12110462 22438439 PMC3309802

[7] Baum T, C Karampinos D, Liebl H, J Rummeny E, Waldt S, S Bauer J. High-resolution bone imaging for osteoporosis diagnostics and therapy monitoring using clinical MDCT and MRI. Current medicinal chemistry. 2013;20(38):4844–4852. 10.2174/09298673113206660279 24083607

[8] Krug R, Burghardt AJ, Majumdar S, Link TM. High-resolution imaging techniques for the assessment of osteoporosis. Radiologic Clinics of North America. 2010;48(3):601–621. 10.1016/j.rcl.2010.02.015 20609895 PMC2901255

[9] Baum T, Carballido-Gamio J, Huber M, Müller D, Monetti R, Räth C, et al Automated 3D trabecular bone structure analysis of the proximal femur - prediction of biomechanical strength by CT and DXA. Osteoporosis international. 2010;21(9):1553–1564. 10.1007/s00198-009-1090-z 19859642 PMC2912724

[10] Mawatari T, Miura H, Hamai S, Shuto T, Nakashima Y, Okazaki K, et al Vertebral strength changes in rheumatoid arthritis patients treated with alendronate, as assessed by finite element analysis of clinical computed tomography scans: a prospective randomized clinical trial. Arthritis & Rheumatism. 2008;58(11):3340–3349. 10.1002/art.23988 18975334

[11] Keaveny TM, Hoffmann PF, Singh M, Palermo L, Bilezikian JP, Greenspan SL, et al Femoral bone strength and its relation to cortical and trabecular changes after treatment with PTH, alendronate, and their combination as assessed by finite element analysis of quantitative CT scans. Journal of Bone and Mineral Research. 2008;23(12):1974–1982. 10.1359/JBMR.080805 18684084 PMC2686921

[12] Keaveny TM. Biomechanical computed tomography—noninvasive bone strength analysis using clinical computed tomography scans. Annals of the New York Academy of Sciences. 2010;1192(1):57–65. 10.1111/j.1749-6632.2009.05348.x 20392218

[13] Graeff C, Timm W, Nickelsen TN, Farrerons J, Marín F, Barker C, et al Monitoring Teriparatide-Associated Changes in Vertebral Microstructure by High-Resolution CT In Vivo: Results From the EUROFORS Study. Journal of Bone and Mineral Research. 2007;22(9):1426–1433. 10.1359/jbmr.070603 17547537

[14] Ziegler A, Köhler T, Proksa R. Noise and resolution in images reconstructed with FBP and OSC algorithms for CT. Medical physics. 2007;34(2):585–598. 10.1118/1.2409481 17388176

[15] Noël PB, Renger B, Fiebich M, Münzel D, Fingerle AA, Rummeny EJ, et al Does iterative reconstruction lower CT radiation dose: evaluation of 15,000 examinations. PloS one. 2013;8(11):e81141 10.1371/journal.pone.0081141 24303035 PMC3841128

[16] Noël PB, Fingerle AA, Renger B, Münzel D, Rummeny EJ, Dobritz M. Initial performance characterization of a clinical noise–suppressing reconstruction algorithm for mdct. American Journal of Roentgenology. 2011;197(6):1404–1409. 10.2214/AJR.11.6907 22109296

[17] Noël PB, Köhler T, Fingerle AA, Brown KM, Zabic S, Münzel D, et al Evaluation of an iterative model–based reconstruction algorithm for low-tube-voltage (80 kVp) computed tomography angiography. Journal of Medical Imaging. 2014;1(3):033501–033501. 10.1117/1.JMI.1.3.033501 26158054 PMC4478879

[18] Marin D, Nelson RC, Schindera ST, Richard S, Youngblood RS, Yoshizumi TT, et al Low-tube-voltage, high-tube-current multidetector abdominal ct: Improved image quality and decreased radiation dose with adaptive statistical iterative reconstruction algorithm?initial clinical experience 1. Radiology. 2009;254(1):145–153. 10.1148/radiol.09090094 20032149

[19] Hara AK, Paden RG, Silva AC, Kujak JL, Lawder HJ, Pavlicek W. Iterative reconstruction technique for reducing body radiation dose at CT: feasibility study. American Journal of Roentgenology. 2009;193(3):764–771. 10.2214/AJR.09.2397 19696291

[20] Fessler JA. Statistical image reconstruction methods for transmission tomography. Handbook of Medical Imaging. 2000;2:1–70.

[21] Erdogan H. Statistical image reconstruction algorithms using paraboloidal surrogates for PET transmission scans. The University of Michigan; 1999.

[22] Adams JE. Quantitative computed tomography. European journal of radiology. 2009;71(3):415–424. 10.1016/j.ejrad.2009.04.074 19682815

[23] Baum T, Gräbeldinger M, Räth C, Garcia EG, Burgkart R, Patsch JM, et al Trabecular bone structure analysis of the spine using clinical MDCT: can it predict vertebral bone strength? Journal of bone and mineral metabolism. 2014;32(1):56–64. 10.1007/s00774-013-0465-6 23604586

[24] Parfitt AM, Drezner MK, Glorieux FH, Kanis JA, Malluche H, Meunier PJ, et al Bone histomorphometry: standardization of nomenclature, symbols, and units: report of the ASBMR Histomorphometry Nomenclature Committee. Journal of bone and mineral research. 1987;2(6):595–610. 10.1002/jbmr.5650020617 3455637

[25] Majumdar S, Genant H, Grampp S, Newitt D, Truong VH, Lin J, et al Correlation of trabecular bone structure with age, bone mineral density, and osteoporotic status: in vivo studies in the distal radius using high resolution magnetic resonance imaging. Journal of Bone and Mineral Research. 1997;12(1):111–118. 10.1359/jbmr.1997.12.1.111 9240733

[26] Chevalier Y, Charlebois M, Pahr D, Varga P, Heini P, Schneider E, et al A patient-specific finite element methodology to predict damage accumulation in vertebral bodies under axial compression, sagittal flexion and combined loads. Computer methods in biomechanics and biomedical engineering. 2008;11(5):477–487. 10.1080/10255840802078022 18608338

[27] Dall’Ara E, Pahr D, Varga P, Kainberger F, Zysset P. QCT-based finite element models predict human vertebral strength in vitro significantly better than simulated DEXA. Osteoporosis International. 2012;23(2):563–572. 10.1007/s00198-011-1568-3 21344244

[28] Muenzel D, Koehler T, Brown K, Žabić S, Fingerle AA, Waldt S, et al Validation of a Low Dose Simulation Technique for Computed Tomography Images. PLoS ONE. 2014 9;9(9):e107843 Available from: http://dx.doi.org/10.1371%2Fjournal.pone.0107843 10.1371/journal.pone.0107843 25247422 PMC4172631

[29] Žabić S, Wang Q, Morton T, Brown KM. A low dose simulation tool for CT systems with energy integrating detectors. Medical physics. 2013;40(3):031102 10.1118/1.4789628 23464282

[30] McCollough CH, Chen GH, Kalender W, Leng S, Samei E, Taguchi K, et al Achieving routine submillisievert CT scanning: report from the summit on management of radiation dose in CT. Radiology. 2012;264(2):567–580. 10.1148/radiol.12112265 22692035 PMC3401354

[31] Yu L, Zou Y, Sidky EY, Pelizzari CA, Munro P, Pan X. Region of interest reconstruction from truncated data in circular cone-beam CT. Medical Imaging, IEEE Transactions on. 2006;25(7):869–881. 10.1109/TMI.2006.872329 16827488

[32] Kolditz D, Kyriakou Y, Kalender WA. Volume-of-interest (VOI) imaging in C-arm flat-detector CT for high image quality at reduced dose. Medical physics. 2010;37(6):2719–2730. 10.1118/1.3427641 20632582

[33] Schafer S, Noël PB, Walczak AM, Hoffmann KR. Filtered region of interest cone-beam rotational angiography. Medical physics. 2010;37(2):694–703. 10.1118/1.3284540 20229879

[34] Schlomka J, Roessl E, Dorscheid R, Dill S, Martens G, Istel T, et al Experimental feasibility of multi-energy photon-counting K-edge imaging in pre-clinical computed tomography. Physics in medicine and biology. 2008;53(15):4031 10.1088/0031-9155/53/15/002 18612175

[35] Taguchi K, Iwanczyk JS. Vision 20/20: Single photon counting x-ray detectors in medical imaging. Medical physics. 2013;40(10):100901 10.1118/1.4820371 24089889 PMC3786515

[36] Nasirudin RA, Mei K, Panchev P, Fehringer A, Pfeiffer F, Rummeny EJ, et al Reduction of Metal Artifact in Single Photon-Counting Computed Tomography by Spectral-Driven Iterative Reconstruction Technique. PLoS ONE. 2015 5;10(5):e0124831 Available from: http://dx.doi.org/10.1371%2Fjournal.pone.0124831 10.1371/journal.pone.0124831 25955019 PMC4425555

[37] Kopp FK, Baum T, Nasirudin RA, Mei K, Garcia EG, Burgkart R, et al Effect of low-dose CT and iterative reconstruction on trabecular bone microstructure assessment. In: SPIE Medical Imaging. International Society for Optics and Photonics; 2016 p. 97881R–97881R. 10.1371/journal.pone.0159903 PMC4957801 27447827

