# Supplementary material for: Effect of Low-Dose MDCT and Iterative Reconstruction on Trabecular Bone Microstructure Assessment
Source: PLoS One. 2016 Jul 22;11(7):e0159903. doi: 10.1371/journal.pone.0159903 (PMC4957801; doi:10.1371/journal.pone.0159903)
Supplement: S1 Table — (PDF) [file pone.0159903.s001.pdf]

S1 Table. Abbreviations and acronyms.

Abbreviation	Definition
MDCT	Multi detector computed tomography
FBP	Filtered backprojection
SIR	Statistical iterative reconstruction
DXA	Dual-energy X-ray absorptiometry
BMD	Bone mineral density
FRAX	Fracture Risk Assessment Tool
MRI	Magnetic resonance imaging
hr-pQCT	High-resolution peripheral quantitative computed tomography
SD	Standard-dose
LD	Low-dose
β	Regularization strength
δ	Regularization threshold
SIR w/o reg.	SIR performed without regularization
BV/TV	Bone volume divided by total volume
TbN	Trabecular number
TbSp	Trabecular spacing
TbTh	Trabecular thickness
app.	Parameters are labeled as apparent values
FD	Fractal dimension
FL	Fracture load
r	Spearman's rank correlation coefficient
SD-FBP	Standard-dose data reconstructed with FBP
LD-FBP	Low-dose data reconstructed with FBP
LD-SIR	Low-dose data reconstructed with SIR
