# Supplementary material for: Effect of Low-Dose MDCT and Iterative Reconstruction on Trabecular Bone Microstructure Assessment
Source: PLoS One. 2016 Jul 22;11(7):e0159903. doi: 10.1371/journal.pone.0159903 (PMC4957801; doi:10.1371/journal.pone.0159903)
Supplement: S2 Table — Vertebrae with the same 3-digit number are from the same donor. The FD parameter value for LD-SIR β = 0.1, δ = 0.0001 of vertebra OPS004_2 could not be determined. The correlation FD vs. FL for LD-SIR β = 0.1, δ = 0.0001 was computed without consideration of vertebra OPS004_2. (PDF) [file pone.0159903.s002.pdf]

S2 Table. Measured trabecular bone microstructure parameters and fracture load for each vertebra. Vertebrae with the same 3-digit number are from the same donor. The FD parameter value for LD-SIR β=0.1, δ=0.0001 of vertebra OPS004_2 could not be determined. The correlation FD vs. FL for LD-SIR β=0.1, δ=0.0001 was computed without consideration of vertebra OPS004_2. BMD was measured for SD-FBP. It is added to each section to provide a better overview.

SD-FBP

ID	Fracture Load [N]	BMD [mg/cm^3]	app.BV/TV	app.TbN [mm^{-1}]	app.TbSp [mm]	app.TbTh [mm]	FD
**OPS003_1**	3181	146,27	0,610	0,997	0,391	0,613	1,608
**OPS003_2**	3991	164,86	0,547	1,028	0,441	0,533	1,615
**OPS003_3**	3719	165,21	0,522	1,031	0,464	0,507	1,575
**OPS003_4**	4147	174,53	0,564	1,045	0,418	0,541	1,619
**OPS004_1**	1212	69,04	0,270	0,806	0,922	0,334	1,484
**OPS004_2**	1912	84,16	0,283	0,830	0,890	0,338	1,477
**OPS004_3**	1704	94,14	0,256	0,766	0,986	0,334	1,457
**OPS004_4**	1853	111,10	0,352	0,906	0,717	0,389	1,534
**OPS005_1**	1951	133,40	0,397	0,988	0,613	0,402	1,507
**OPS005_2**	1990	120,74	0,389	0,991	0,620	0,393	1,494
**OPS005_3**	2513	137,11	0,453	0,979	0,559	0,464	1,529
**OPS005_4**	3141	118,56	0,360	0,904	0,710	0,399	1,548
**Spearman correlation vs. FL**		0,92	0,90	0,88	-0,90	0,91	0,89

LD-FBP

ID	Fracture Load [N]	BMD [mg/cm^3]	app.BV/TV	app.TbN [mm^{-1}]	app.TbSp [mm]	app.TbTh [mm]	FD
**OPS003_1**	3181	146,27	0,543	1,164	0,393	0,466	1,611
**OPS003_2**	3991	164,86	0,516	1,126	0,430	0,458	1,621
**OPS003_3**	3719	165,21	0,536	1,061	0,438	0,506	1,585
**OPS003_4**	4147	174,53	0,529	1,106	0,427	0,478	1,622
**OPS004_1**	1212	69,04	0,352	1,002	0,649	0,351	1,560
**OPS004_2**	1912	84,16	0,359	1,000	0,645	0,358	1,551
**OPS004_3**	1704	94,14	0,355	1,029	0,629	0,345	1,566
**OPS004_4**	1853	111,10	0,417	1,056	0,553	0,394	1,590
**OPS005_1**	1951	133,40	0,431	1,074	0,531	0,402	1,538
**OPS005_2**	1990	120,74	0,422	1,080	0,536	0,391	1,543
**OPS005_3**	2513	137,11	0,471	1,082	0,490	0,436	1,563
**OPS005_4**	3141	118,56	0,391	1,064	0,574	0,367	1,586
**Spearman correlation vs. FL**		0,92	0,85	0,77	-0,85	0,85	0,62

LD-SIR w/o reg.

ID	Fracture Load [N]	BMD [mg/cm^3]	app.BV/TV	app.TbN [mm^{-1}]	app.TbSp [mm]	app.TbTh [mm]	FD
**OPS003_1**	3181	146,27	0,481	1,078	0,483	0,445	1,572
**OPS003_2**	3991	164,86	0,524	1,047	0,455	0,501	1,613
**OPS003_3**	3719	165,21	0,464	0,997	0,540	0,465	1,544
**OPS003_4**	4147	174,53	0,466	1,036	0,516	0,451	1,595
**OPS004_1**	1212	69,04	0,289	0,827	0,875	0,348	1,494
**OPS004_2**	1912	84,16	0,291	0,805	0,913	0,356	1,483
**OPS004_3**	1704	94,14	0,234	0,735	1,069	0,317	1,449
**OPS004_4**	1853	111,10	0,313	0,818	0,844	0,384	1,484
**OPS005_1**	1951	133,40	0,376	0,928	0,675	0,406	1,472
**OPS005_2**	1990	120,74	0,360	0,947	0,678	0,380	1,464
**OPS005_3**	2513	137,11	0,361	0,911	0,706	0,396	1,463
**OPS005_4**	3141	118,56	0,386	0,945	0,658	0,407	1,558
**Spearman correlation vs. FL**		0,92	0,93	0,87	-0,90	0,92	0,69

LD-SIR β=0.1, δ=0.0001

ID	Fracture Load [N]	BMD [mg/cm^3]	app.BV/TV	app.TbN [mm^{-1}]	app.TbSp [mm]	app.TbTh [mm]	FD
**OPS003_1**	3181	146,27	0,470	0,693	0,778	0,677	1,460
**OPS003_2**	3991	164,86	0,540	1,032	0,446	0,525	1,616
**OPS003_3**	3719	165,21	0,433	0,604	0,948	0,718	1,407
**OPS003_4**	4147	174,53	0,383	0,579	1,075	0,660	1,439
**OPS004_1**	1212	69,04	0,100	0,161	13,856	0,602	0,934
**OPS004_2**	1912	84,16	0,179	0,232	9,036	0,456	NaN
**OPS004_3**	1704	94,14	0,041	0,097	17,081	0,386	0,664
**OPS004_4**	1853	111,10	0,352	0,862	0,756	0,409	1,513
**OPS005_1**	1951	133,40	0,202	0,363	2,464	0,550	1,158
**OPS005_2**	1990	120,74	0,150	0,348	2,671	0,417	1,070
**OPS005_3**	2513	137,11	0,339	0,558	1,212	0,599	1,326
**OPS005_4**	3141	118,56	0,333	0,479	1,465	0,677	1,389
**Spearman correlation vs. FL**		0,92	0,81	0,67	-0,67	0,58	0,65

LD-SIR β=0.001, δ=0.0001

ID	Fracture Load [N]	BMD [mg/cm^3]	app.BV/TV	app.TbN [mm^{-1}]	app.TbSp [mm]	app.TbTh [mm]	FD
**OPS003_1**	3181	146,27	0,481	1,062	0,490	0,452	1,570
**OPS003_2**	3991	164,86	0,517	1,034	0,467	0,501	1,610
**OPS003_3**	3719	165,21	0,461	0,975	0,556	0,473	1,541
**OPS003_4**	4147	174,53	0,452	1,018	0,538	0,445	1,585
**OPS004_1**	1212	69,04	0,272	0,770	0,970	0,350	1,467
**OPS004_2**	1912	84,16	0,301	0,813	0,882	0,366	1,489
**OPS004_3**	1704	94,14	0,198	0,639	1,299	0,307	1,392
**OPS004_4**	1853	111,10	0,322	0,823	0,827	0,392	1,491
**OPS005_1**	1951	133,40	0,351	0,883	0,738	0,398	1,445
**OPS005_2**	1990	120,74	0,369	0,944	0,670	0,391	1,470
**OPS005_3**	2513	137,11	0,400	0,939	0,640	0,427	1,494
**OPS005_4**	3141	118,56	0,344	0,885	0,754	0,387	1,529
**Spearman correlation vs. FL**		0,92	0,90	0,91	-0,91	0,84	0,89
